# Think Aloud Protocol Applied in Naturalistic Driving for Driving Rules Generation

**DOI:** 10.3390/s20236907

**Published:** 2020-12-03

**Authors:** Borja Monsalve, Nourdine Aliane, Enrique Puertas, Javier Fernández Andrés

**Affiliations:** 1 Science, Computing and Technology Department, Universidad Europea de Madrid, 28670 Madrid, Spain; enrique.puertas@universidadeuropea.es; 2 Industrial and Aerospace Engineering Department, Universidad Europea de Madrid, 28670 Madrid, Spain; nourdine.aliane@universidadeuropea.es (N.A.); javier.fernandez@universidadeuropea.es (J.F.A.)

**Keywords:** autonomous driving, naturalistic driving, think aloud protocol, driver behavior, rule generation, cognitive process

## Abstract

Understanding naturalistic driving in complex scenarios is an important step towards autonomous driving, and several approaches have been adopted for modeling driver’s behaviors. This paper presents the methodology known as “Think Aloud Protocol” to model driving. This methodology is a data-gathering technique in which drivers are asked to verbalize their thoughts as they are driving which are then recorded, and the ensuing analysis of the audios and videos permits to derive driving rules. The goal of this paper is to show how think aloud methodology is applied in the naturalistic driving area, and to demonstrate the validity of the proposed approach to derive driving rules. The paper presents, firstly, the background of the think aloud methodology and then presents the application of this methodology to driving in roundabouts. The general deployment of this methodology consists of several stages: driver preparation, data collection, audio and video processing, generation of coded transcript files, and the generation of driving rules. The main finding of this study is that think aloud protocol can be applied to naturalistic driving, and even some potential limitations as discussed in the paper, the presented methodology is a relatively easy approach to derive driving rules.

## 1. Introduction

Autonomous driving has been a very active field of research in recent years [1,2]. According to the Automated and Autonomous Driving of the National Highway Traffic Administration (NHTSA), the industry is already far from the fifth level of “full automation”, where vehicles are capable of performing all the driving functions under all conditions [3]. One of the main benefits offered by autonomous cars is the safety. According to the World Health Organization (WHO), about 1.3 million people die on the roads around the World, and about 20–50 million are injured every year [4]. According to NHTSA, 94% of serious car accidents are due to human error, so autonomous vehicles would have the potential to remove this factor from the equation. This would increase the safety for the drivers, passengers, pedestrians, and other vehicles on the road. Other studies like [5] also reveal the economic and societal benefits of this industry, without mentioning the productivity, pollution, and quality of life problems derived from traffic jams in the cities.

Different techniques have been used to model car behavior. Most studies have been based on simulated environments [6,7,8] or instrumented cars [9]. The first group allow researchers to reproduce concrete situations on demand, and in the second case the behavior attends to real environment. Driver models have also been developed to analyze many aspects of the driving process. Initial studies focused on issues related to low-level control behavior, like curve negotiation [10] or steering control [11]. Later, these techniques were combined with other high-level models to develop integrated driver models like [12] or [13], where a cognitive architecture is proposed to model the driver behavior.

Other methodologies used to analyze and model driver’s behavior are based on naturalistic driving, where data related to the car, the driver, and the environment of the route are gathered [14] for later analysis. These kinds of studies have been mainly used related to driving safety, for example for collision warning [15], safety events related to weather conditions [16], or for measuring time to collision at braking depending on the age and gender of the driver [17]. To be able to create a true-life model, these studies configure a nonintrusive driving environment to gather data during well-known routes for the driver [18]. There is no supervision during the process, so the subject receives no in-situ instructions that might affect his or her usual behavior. Compared to more traditional strategies, naturalistic driving studies have several advantages [19]. One of the most important is the opportunity to understand a driver’s real behavior more broadly, as they are based on real situations during daily routes. The main disadvantage is just that lack of control over the experiment itself, as a result of this absence of interventionism.

Another promising field of research to model driving is related to the use of think aloud protocol. This technique has been widely used in many fields to analyze problem solving, such as in thinking study [20], in translation research [21], or user interface design [22]. This methodology is also used in the field of autonomous driving. For example, in [23] the thinking aloud protocol is used to obtain deeper insight into the interaction of drivers with semiautonomous vehicles (level-2 automation) measuring the degree acceptance of users of different age groups. The same methodology is used in [24], as part of the knowledge extraction process of a system, to train drivers how to interact with semiautonomous vehicles. In [25], this methodology is also used to better understand the anticipation capabilities of drivers, with the aim of increasing safety in any situation in autonomous environments. However, there does not seem to be previous work in which the think aloud protocol is used for deriving driving rules in specific traffic scenarios.

In this study, the use of the think aloud protocol is proposed aimed at extracting rules modeling the driver’s behavior following naturalistic driving, where the main task of the driver consists in the verbalization of his/her thoughts that occurred during the driving task. Afterwards, the recorded talks will be analyzed with the goal of extracting driving rules.

The rest of the paper is organized as follows: first the think aloud protocol is explained, and how this technique is applied within naturalistic driving context to model driver behavior. The next section presents the experimental environment, the data collection process, as well as the management of this information. Afterwards, the cognitive process applied to data extraction and analysis as well as the derivation of driving rules are explained. The following section is dedicated to maneuvers within roundabouts. Finally, conclusions and future work are drawn in the last section.

## 2. What Is the Think Aloud Protocol?

When a person is going to carry out a cognitive process or carry out an action, the cognitive system collects the information through the senses (sight, hearing, touch, etc.) and deposits that information in the working memory, also known as short term memory. Then the brain collects the information from working memory, processes it, and executes the next action to take. This action is also stored in the working memory before being transferred to the motor system that will execute the action, such as moving the neck to look to the side. Therefore, if we could know the content of working memory, we can know which inputs and outputs are involved in a cognitive process when performing a certain action.

The think aloud protocol is based on the theory that people are able to verbalize the linguistic content of working memory, and its goal is to capture what the content of a person’s working memory is when they perform a task or solve a problem.

**Figure 1:**
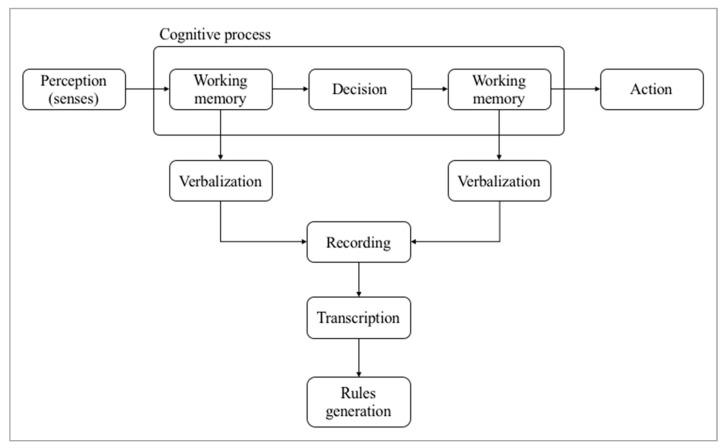
Main steps in the think aloud method.

Think aloud protocol is a methodology where participants are asked to talk about the task they are working at [26], saying out loud what they are looking at, thinking about and doing, in order to extract the content of the working memory, to capture inputs and outputs involved in a task, and aiming to create a model that explains the behavior of the subject. This strategy was proposed by Newel and Simon in 1972 [27] to generate reasoning models based on human behavior. The thoughts expressed following this technique are recorded, so later can be transcribed and then analyzed following a set of preconfigured categories depending on the domain and the kind of tasks of the study. Think aloud protocol is based on the idea that people are able to verbalize the linguistic content stored in their working memory, which can be expressed with words. This strategy has been used to analyze problem solving activities in many fields, such as cognitive psychology [20], translation processes [21,28,29], to analyze the writing process [30] or user interface and evaluation of usability in computer science [22,31], among others. For example, in [32], researchers present the evaluation of a computer system design based on think aloud protocol. For this purpose, they made a selection of nine different system designers attending to a variety of roles. All of them had previous experience in the think aloud method, and faced different tests of software application. The interviews were recorded and later fully transcribed, to be analyzed in detail after that. This study showed the value of the feedback obtained using this technique, that helped designers to develop better systems. Figure 1 shows the steps involved in the think aloud protocol.

The methodology has two main stages: namely data collection, and model building and analysis. Furthermore, as suggested by Ericson and Simon [33], the first stage is divided into three levels.

In level 1 reports, direct vocalization is produced as a result of the verbalization of the ideas stored in the subject’s short-term memory. These kinds of reports are generated by simple instructions that only imply thinking aloud and demand no extra effort by the subject. These types of protocols are only useful when the data stored in the working memory is mostly linguistic information. Most of the concepts belong to this first level, where perceptions such as smell or taste are difficult to verbalize. Thus, a translation of these abstract concepts to linguistic expressions are needed. Unfortunately, the concepts that drivers observe and the actions they execute do not always belong to linguistic information, and their representation in memory is not directly verbalizable, such as traffic signs, vehicle indicators, etc. In such case, subjects will have to think about the necessary vocabulary to represent the concept clearly. This will take place during driving activities.

Level 2 takes place when the task requires processing extra details. To verbalize them, the subject needs to manage descriptions about the content stored in his working memory, but without searching into his long-term memory. The time necessary to develop the task used to be longer although cognitive process is not affected.

Finally, in level 3, the task demands conscient control. The cognitive process needs to work with data that cannot be found in the working memory, so it requires different introspection grades. This tasks require more time to be solved, but the “overload” introduced by the speech does not modify the cognitive process itself [33].

In this work, the combination of the think aloud protocol with naturalistic driving to extract driving rules is proposed. The idea consists of analyzing the records of real driving as well as other data derived from naturalistic driving. Furthermore, the study is focused on a roundabout driving scenario, where the driving task involves carrying out several steps:

- Perceive the environment.
- Decision making after a cognitive process.
- Execute the planned action.
- Modify the environment.

**Figure 2:**
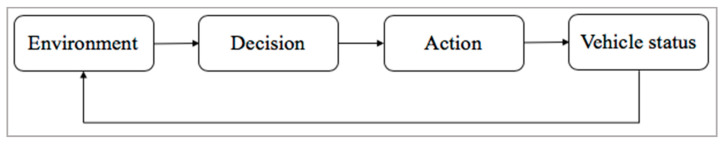
Driving behavior.

The driver perceives the environment to extract data that supports the decisions to be taken. These new actions executed, such as accelerating, braking, etc., lead to changes in the vehicle’s states, and so also does the environment, which generates a new cycle as shown in Figure 2.

## 3. Experimental Setting and Data Collection

**Figure 3:**
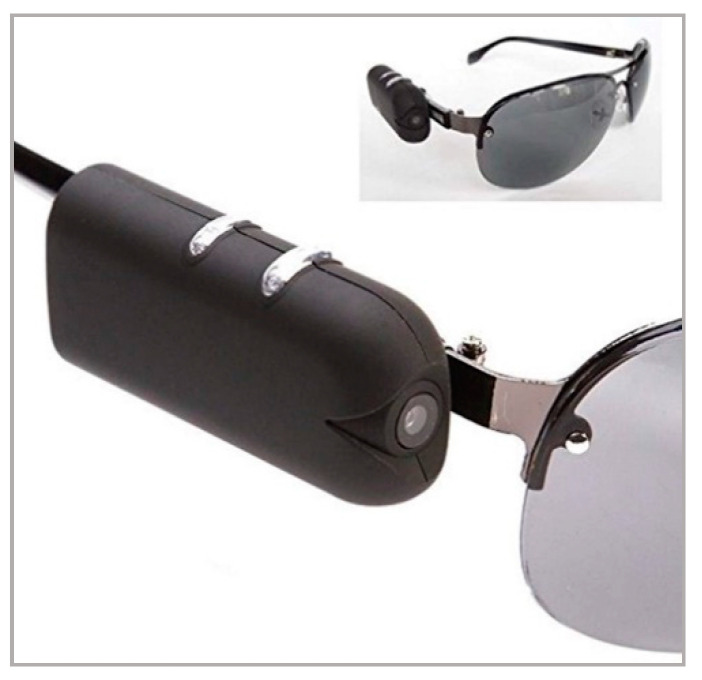
Portable camera.

The data needed for the present study were mainly audio of driver’s verbalization as well as the videos of the driving scenes. The basic instrumentation used in the data collection process consists of a glasses mounted camera (as shown in Figure 3), and smartphone mounted on the vehicle dashboard for recording simultaneously two additional videos: the front view of the vehicle for saving the driving scenes and the rear view for recording the driver actions.

The next step consists of generating transcript text files from the videos and audios. The text was then analyzed along with the video images. The actions indicated by the driver were coded. In many cases, due to the overload of stimuli and actions, evident actions were added to the transcript files, based on the video contents, and sometimes by guessing the driver intentions because the corresponding actions verbalizations were omitted. However, for the transcript files and their subsequent analysis to be useful, verbalizations should be effective.

**Figure 4:**
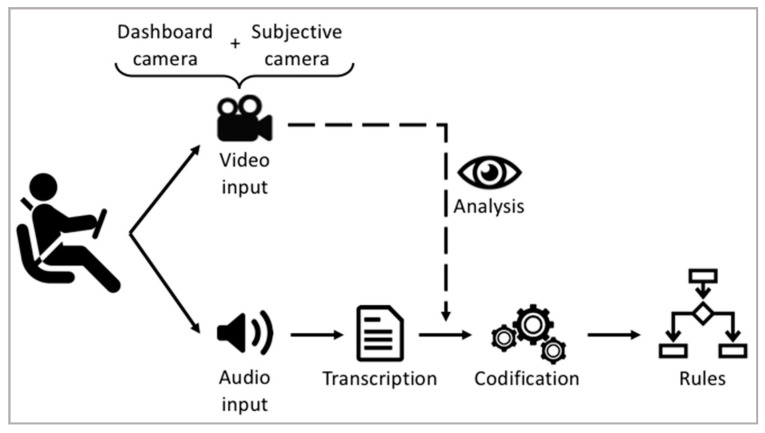
Data acquisition and analysis process.

The steps of data acquisition and transcript text analysis processes are summarized in Figure 4.

To ensure data recording close to naturalistic driving as possible and avoiding undesirable behaviors, the driver was prepared with a prior training for adhering to a protocol for a reliable and effective verbalization. The training stage consisted of a few routes where the driver was assisted by the research group to help him to express aloud what he was perceiving and what he was executing. It is worth to point out that, from the analyzer point of view, driver’s emotions or opinions were not targeted. Only the verbalization of the perceptions and actions related to driving were taken into account. Some example could be, for instance, acceleration, brake, “I look in the rearview mirror”, “I see a car approaching quickly from the left”, etc. The issues related to concurrent actions were solved by verbalizing those using short sequential sentences. After some journeys, the driver reached to put the focus on his driving.

## 4. Cognitive Process

Cognitive process in the think aloud protocol refers to the process of data extraction and the subsequent analysis for building and validating a specific model. As far as the think aloud protocol applied to driver modeling is concerned, its main goal consists in extracting a set of driving rules to be implemented in an autonomous vehicle. The process focuses on the observation and knowledge extraction of how the subject acts when facing the different actions involved when trying to solve a concrete task.

Considering the Stop maneuver as an example, three main steps could be identified:

1. Stop the vehicle.
2. Analyze the circulation.
3. Resume the route.

Depending on the goal of the process, the abstraction level of the steps could be required to be more detailed, when performing the previously mentioned maneuver. In this maneuver, the vehicle’s direction and speed were taken as input data as well as the direction by which we wanted to continue after making the stop. Additionally, step 2 “Analyze the circulation” could be detailed into several subtasks depending on the exit direction:

- Check traffic coming from the left.
- Check traffic coming from the right.
- Check traffic coming from the front.

The transcript text files processing was performed following Ronald Hamel’s approach described in [34] by creating a set of labels for categorizing the actions with two main input stimuli, external sounds and external visual information (labeled as Sight and Sound), that the driver needs to process driving. On the actuation side, a normal driver uses his arms and legs as actuators to manipulate the controls of the vehicle and modify its state. According to this, a set of possible actions to perform were defined, based on the actuators or sensors involved in the task.

**Figure 5:**
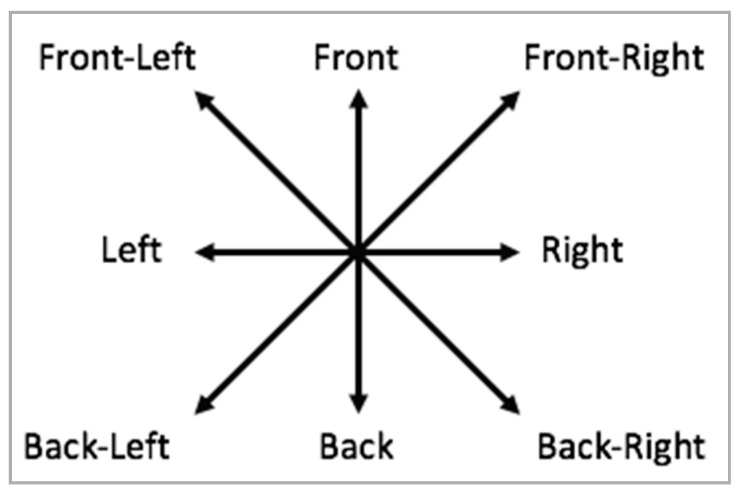
Direction of input stimuli.

Furthermore, eight possible directions from where visual stimuli are captured were defined (see Figure 5). Concerning external sounds, they were considered two cases according to the location of the sound source: inside and outside the vehicle. Finally, a table coding the different situations was created. For example, if the driver was looking diagonally between the front and the left according to the travel direction (Front-Left view), it was coded as “FLV”. The codes corresponding to sound and sight stimuli and their descriptions are given in Table 1.

The codification of the different actions was organized in two categories linked to hands and feet. The main actions to be taken into account when a vehicle undertakes a roundabout are hands for steering wheel, the gear shift lever, or the blinkers, and feet for acting on the pedals. This set of actions and their codes were determined by the kind of vehicle used in the experiments, such as a car with manual or automatic gearbox. Table 2 gives the codes of hands and feet actions for a manual gear car.

## 5. Case of a Roundabout Maneuver

**Figure 6:**
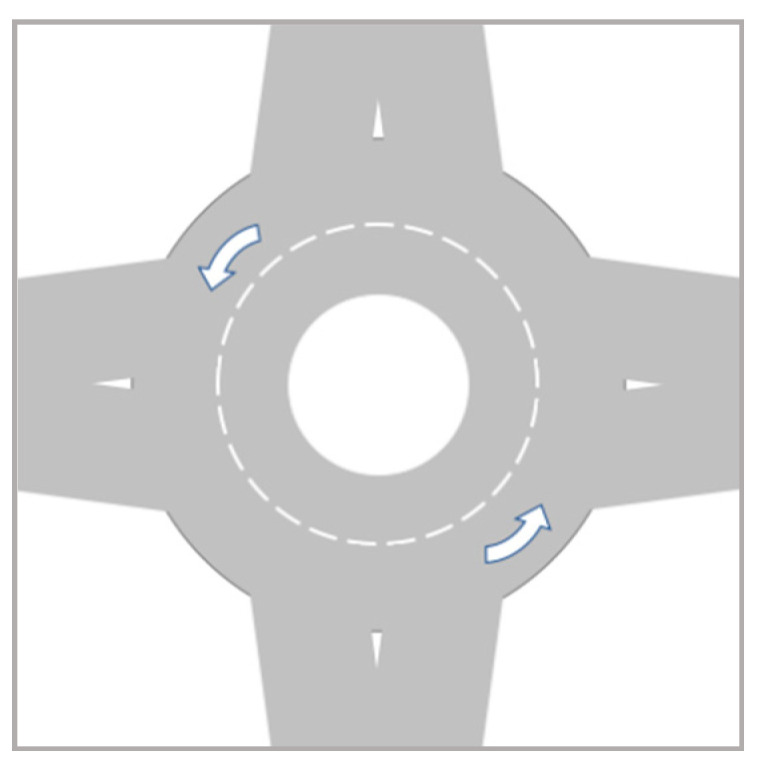
Standard roundabout.

According to the traffic and road safety regulations in Spain [35], a roundabout represents an intersection in the road where different sections connect around a central island (see Figure 6). In Spain, vehicles approaching roundabouts do not have preference over those being already inside them. Thus, the predefined right-side preference rule changes in this situation.

The study carried out in this article was limited to modeling driving in roundabouts, since they are considered as a complex traffic scenario [36,37,38]. To capture the essence of maneuvers within roundabouts, and from driver attention point of view, according to the approach applied in [39], roundabouts are divided into three sections: approaching roundabouts, driving inside applying the priority rules, and leaving them.

To validate the proposed methodology, the participation of one driver was enough to carry out the experiments. In this case, the participant for recording the audios and the videos recording was a 44-year-old male driver with 25 years driving experience, and he used his own manual gearbox vehicle.

On the other hand, in order to avoid prolonged stress while driving, journeys of 10–15 min driving were chosen. The same driver, using his own car, drove along a route with five roundabouts 10 times. A total of 50 audio recordings and videos were obtained. Finally, the frames corresponding to roundabouts stretches were extracted for their subsequent analysis.

Afterwards, these new videos were edited and composed so that their visualization would be straightforward and effective, and were automatically processed with dictation software for creating a first transcript version, which was supervised to fix errors if it was needed. Finally, the transcription of the audio was then stored in a text file.

**Figure 7:**
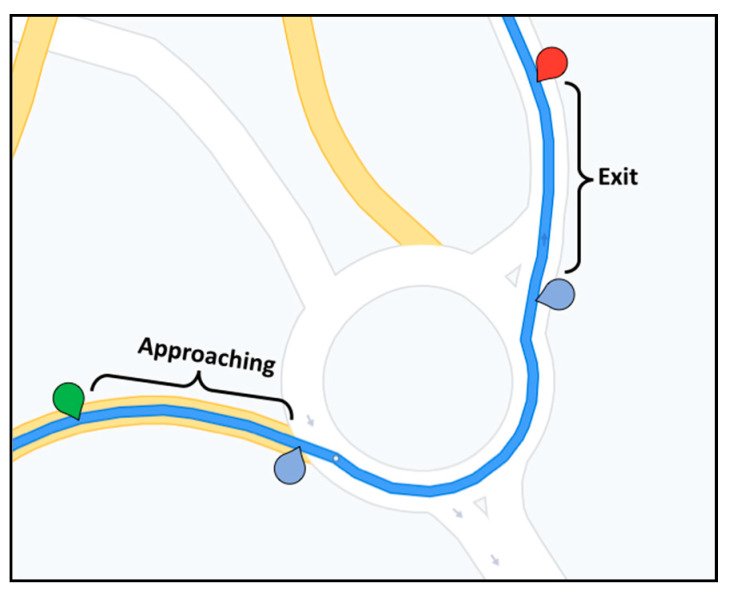
Roundabout example.

**Figure 8:**
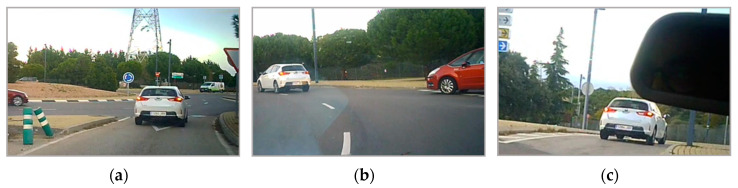
Three frames of each section of the maneuver: (**a**) approaching, (**b**) inside, and (**c**) exiting the roundabout.

Later, some additional information were added to the generated transcription files, such as defining labels for the start and the end of roundabouts, their durations as well as including labels corresponding to actions verbalized by the driver, using the codes defined in Table 1 and Table 2. Evident actions shown in the video but omitted by the driver were also included. Labeled transcription was then analyzed to study the input and output of each situation, to generate pseudocode rules that can explain the behavior of the driver when facing a roundabout. As an example, a transcription fragment of a maneuver in the roundabout (see Figure 7) is shown in Table 3. The different actions verbalized by the driver, but also those raised from the analysis of the videos are indicated in the text. Figure 8 shows some video frames of the roundabout of the example taken with the subjective camera.

After the analysis of these labeled transcriptions files, the steps followed by the driver during the maneuver were converted to pseudocode. Following is an example of a pseudocode generated from the transcription files of Table 3 of the previous example.
IF approaching_roundabout THEN   reduce speed   WHILE other_vehicle_inside_roundabout THEN     Stop   speed(increase)   speed(hold)   direction(right)   blinker(left)   direction(left)   blinker(right)   direction(right)   speed(increase)

These rules were generated in a supervised process by the researchers. As they were specified at high level, is easy to understand for a human being, and this makes them useful to analyze the cognitive process of the studied task. However, in order to be incorporated into an autonomous driving vehicle, they will require extra processing to make them more specific, depending on the detail required by the system. The resulting rules are strongly dependent on the driver behavior and the way he or she performs the studied roundabouts. Thus, veteran or a more aggressive driver could help us to achieve different rules from those derived form a novice or a more cautious one. They are also dependent on very subjective concepts or perceptions, such as what can be understood by “close” or “fast” when executing the maneuver, regardless of what may be marked by traffic rules or road signs. The rules can be easily validated and adjusted using simulation systems, starting with a reproduction of the roundabouts used for the experiments, and later using new ones to check if they can be generalized to other roundabout types and traffic situations.

This study, however, presents some limitations. For example, the generated driving rules do not consider possible incidents that might occur. However, these kinds of situations may be handled including exceptions management in the generated rules. Another limitation is related to the analysis that is supervised in many stages. This issue can be improved by automating some tasks by the use of speech-to-text translators and the use of automatic transcription processing techniques. Although the paper is focused on roundabouts, the study may be extended to other maneuvers, such as incorporation to highways, intersections, etc. Finally, driving rules derived from think aloud protocol can be assessment using some kind of virtual simulator.

## 6. Conclusions and Future Work

In this paper the think aloud protocol technique in a naturalistic driving environment to model driver behavior was presented, which consists of the driver verbalizing all the performed actions while driving. This work is limited to the study of maneuvers at roundabouts.

The deployment of this technique requires an initial training, permitting drivers to act according to a predefined protocol. This is necessary to analyze and interpret the driving actions properly.

The data of the experiments, obtained mainly from video and audio recordings, were obtained during several trips but carried out by the same driver. Firstly, processing consists of isolating the driving to roundabouts segments, which were then divided into three parts: approaching, inside, and exiting sections. Afterwards, each section was analyzed and coded using a set of predefined actions. The result was a set of enriched texts combining transcriptions and coded actions. These enriched texts were then analyzed for deriving pseudocode rules corresponding to driving.

As future lines of work, and with the aim of minimizing the supervised stages of the process of obtaining driving rules, the first idea consists of using speech-to-text translators and using automatic transcription processing techniques for the detection of keywords related to in driving maneuvers. The second idea deals with obtaining more driving rules using other traffic scenarios, such as incorporation to highways, intersections, etc., and then performing a first assessment of the derived driving rules in the virtual simulator.

## Figures and Tables

**Table 1:** Codes of input stimuli.

Category	Action	Code	Description
Sight	Front view	FV	Look forward in the driving direction
Sight	Front view to the rearview mirror	FV-Mirror	Look through the interior rearview mirror
Sight	Left view	LV	Look left
Sight	Left mirror	LV-Mirror	Look left through the left external mirror
Sight	Front-Left view	FLV	Look diagonally, between front and left
Sight	Right view	RV	Look right
Sight	Right mirror	RV-Mirror	Look left through the right external mirror
Sight	Front-Right view	FRV	Look diagonally, between front and right
Sight	Back view	BV	Look turning head back.
Sight	Back-Left view	BLV	Look back diagonally between left and rear of the vehicle.
Sight	Back-Right view	BRV	Look back diagonally between right and rear of the vehicle.
Sound	Inside sound	IN-S	Sound from inside the vehicle
Sound	Outside sound	OUT-S	Sound coming from outside the vehicle

**Table 2:** Codes of hands and feet actions for a manual gear car.

Category	Action	Code	Description
Hands	Turning steering wheel left	SW-TL-L	Turn the steering wheel to the left to change direction to the left
Hands	Turning steering wheel left	SW-TL-S	Turn the steering wheel to the left to straighten the car and continue straight
Hands	Turning steering wheel right	SW-TR-R	Turn the steering wheel to the right to change direction to the right
Hands	Turning steering wheel right	SW-TR-S	Turn the steering wheel to the right to straighten the car and continue straight
Hands	Gear down	GD	Reduce N gears to decrease speed
Hands	Gear up	GU	Increase N gears to increase speed
Hands	Left blinker light on	LB-ON	Use the turn signal switch to turn the left light blinker on
Hands	Left blinker light off	LB-OFF	Use the turn signal switch to turn the left light blinker off
Hands	Right blinker light on	RB-ON	Use the turn signal switch to turn the left right blinker on
Hands	Right blinker light off	RB-OFF	Use the turn signal switch to turn the left right blinker off
Feet	Activate clutch pedal	G-ON	Activate the clutch pedal to initiate gear changing
Feet	Release clutch pedal	G-OFF	Release the clutch pedal to finish gear changing
Feet	Push throttle pedal	T-ON	Push the throttle further to increase speed
Feet	Hold throttle pedal	T-HOLD	Keep throttle pedal pressed to maintain stable speed
Feet	Release throttle pedal	T-OFF	Release throttle pedal to deaccelerating the vehicle and reduce speed
Feet	Push brake pedal	B-ON	Push brake pedal to reduce speed. It implies having previously released the accelerator
Feet	Release brake pedal	B-OFF	Release the brake pedal to stop braking the car

**Table 3:** Transcriptions of the three sections of the roundabout.

Section	Roundabout Approaching
Duration	20 s
Transcription	“I let the car go slowly <T-OFF>, brake a little <B-ON>. I engage the clutch <G-ON>, I go into third gear <GD>, I take my feet off the pedals <G-OFF>, I apply the brakes again a little bit <B-ON>… I keep braking <B-ON> <SW-TR -R> a red car is coming up behind me <FV-MIRROR>. I shift into second gear <G-ON> <GU>, I release the clutch <G-OFF>, brake <B-ON> because here <SW-TL-S> <LV> there was a car at the roundabout.”
Section	Inside roundabout
Duration	15 s
Transcription	“<LV> Now we are going to go through <T-ON> <SW-TR-R> <T-HOLD> and now I turn on the blinker to the left <LB-ON> <SW-TL-L>, now I turn on the blinker to the right <RB-ON>. And I am going to exit here at the <SW-TR-R> roundabout behind the white car.”
Section	Roundabout exit
Duration	6 s
Transcription	“The blinker turns off <SW-TR-R> <SW-TL-S> <T-ON>, I pass the crosswalk, in that other crosswalk there is no one, so I keep going.”
